# Why do Large Animals Never Actuate Their Jumps with Latch-Mediated Springs? Because They can Jump Higher Without Them

**DOI:** 10.1093/icb/icz145

**Published:** 2019-08-09

**Authors:** Gregory P Sutton, Elizabeth Mendoza, Emanuel Azizi, Sarah J Longo, Jeffrey P Olberding, Mark Ilton, Sheila N Patek

**Affiliations:** 1 School of Life Sciences, University of Lincoln, Lincoln, UK; 2 School of Biological Sciences, University of California, Irvine, CA, USA; 3 Biology Department, Duke University, Durham, NC; 4 Department of Physics, Harvey Mudd College, Claremont, CA

## Abstract

As animals get smaller, their ability to generate usable work from muscle contraction is decreased by the muscle’s force–velocity properties, thereby reducing their effective jump height. Very small animals use a spring-actuated system, which prevents velocity effects from reducing available energy. Since force–velocity properties reduce the usable work in even larger animals, why don’t larger animals use spring-actuated jumping systems as well? We will show that muscle length–tension properties limit spring-actuated systems to generating a maximum one-third of the possible work that a muscle could produce—greatly restricting the jumping height of spring-actuated jumpers. Thus a spring-actuated jumping animal has a jumping height that is one-third of the maximum possible jump height achievable were 100% of the possible muscle work available. Larger animals, which could theoretically use all of the available muscle energy, have a maximum jumping height that asymptotically approaches a value that is about three times higher than that of spring-actuated jumpers. Furthermore, a size related “crossover point” is evident for these two jumping mechanisms: animals smaller than this point can jump higher with a spring-actuated mechanism, while animals larger than this point can jump higher with a muscle-actuated mechanism. We demonstrate how this limit on energy storage is a consequence of the interaction between length–tension properties of muscles and spring stiffness. We indicate where this crossover point occurs based on modeling and then use jumping data from the literature to validate that larger jumping animals generate greater jump heights with muscle-actuated systems than spring-actuated systems.

## Introduction

As animals decrease in size, it is increasingly difficult to generate muscle-driven jumps with high-velocities, heights, or distances (Bobbert 2013). Small animals have short limbs and thus muscles require a high active strain rate to extend the legs fast enough to generate similar jump velocities similar to larger animals (Bobbert 2013). However, the greater a muscle’s active strain rate, the less force (and thus less work) it is able to generate, a property is known as the muscle’s “force–velocity curve” (Hill 1938; Huxley 1957). Jump velocity, and ultimately the height and distance reached by a jumping animal, depends on the work performed by muscles relative to the mass of the animal (Borelli 1680; Bobbert 2013). Because very small animals (weighing <1 g) produce less network relative to their mass, they are unable to use a muscle-actuated system to jump as high as larger animals (Bobbert 2013; Sutton et al. 2016).

Many small animals, like insects, use springs instead of muscles to actuate leg extension, thereby circumventing force–velocity induced reduction of muscle work (Bennet-Clark 1975; Patek et al. 2011). To do this, insects first latch their legs in place and then slowly contract muscles that store muscle work in elastic cuticular structures. By contracting very slowly, the muscle generates work without experiencing losses due to velocity effects. After elastic energy is stored, the latch is released, and the cuticular structures act as springs, recoiling to actuate the legs. During this recoil, nearly 100% of the stored elastic energy propels the animal into the air (Bennet-Clark 1975; Alexander 1995; Patek et al. 2011). As such, for spring-actuated jumpers with an equivalent percentage of body-mass devoted to muscle, jump performance is predicted to be independent of mass (Borelli 1680; Bobbert 2013), resulting in a constant jump height regardless of the insect’s body size (Burrows and Sutton 2008).

Latch-mediated spring-actuated systems are important for small animals in which force–velocity effects lead to large reductions in muscle work. However, non-zero reductions in muscle work also occur in larger animals (Zajac 1989; Bobbert 2013). While some systems can mitigate this by executing a “countermovement” to store some elastic energy and manipulate the force–velocity curve (Pandy and Zajac 1991; Alexander 1995), force–velocity effects on muscle work still affect performance of muscle-actuated jumping animals of all sizes. In contrast, force–velocity effects cause no reduction of muscle work in latch-mediated spring-actuated jumping systems and consequently jump height in these systems is independent of size (Burrows and Sutton 2008). Moreover, simulations of force–velocity effects on jump height have predicted that latch-mediated spring-actuated jumpers would be able to jump higher than an equivalently sized muscle-actuated jumper at all sizes (Alexander 1995), with this effect being smaller, but non-zero, for larger animals. This raises the question, if spring-actuated jumping systems lose no potential work to muscle force–velocity properties, why don’t large animals use them as well?

Here we address this question by directly assessing an incorrect assumption made by previous models of latch-mediated spring-actuated jumping. While it is correct to assume that work done in spring-actuated jumpers is not reduced by velocity effects, the work done in spring-actuated systems is, however, reduced by interactions between the spring stiffness and muscle length–tension properties. While previous models have predicted that the more compliant the spring, the higher the jump (Alexander 1995), more recent models of the interactions between spring stiffness and muscle length–tension properties predict an optimal, intermediate stiffness that maximizes energy storage (Rosario et al. 2016). We demonstrate that even at this optimal spring stiffness, the energy that can be stored in latch-mediated spring-actuated systems is limited to ∼30% of the work that a muscle could generate based on its length–tension properties. In contrast with muscle-actuated jumpers, in which work is limited by force–velocity effects, the energy output of spring-actuated jumpers is limited by length–tension effects.

By using a simple model of a muscle driving a mass, we show how these two effects influence energy output differently, with velocity effects varying with an animal’s size, whereas length–tension effects are independent of size. Because of the different interactions between size and energy output, there is consequently a size-related “crossover point.” Animals larger than this point generate more energy with a muscle-actuated system, and animals smaller than this point generate more energy with a spring-actuated system. Lastly, we show that jump heights of different animals from the published literature are congruent with this new analysis: larger jumping animals reach heights that are over three times greater than latch-mediated spring-actuated jumpers of any size.

## Materials and methods

### Kinetic modeling

**Fig. 1:**
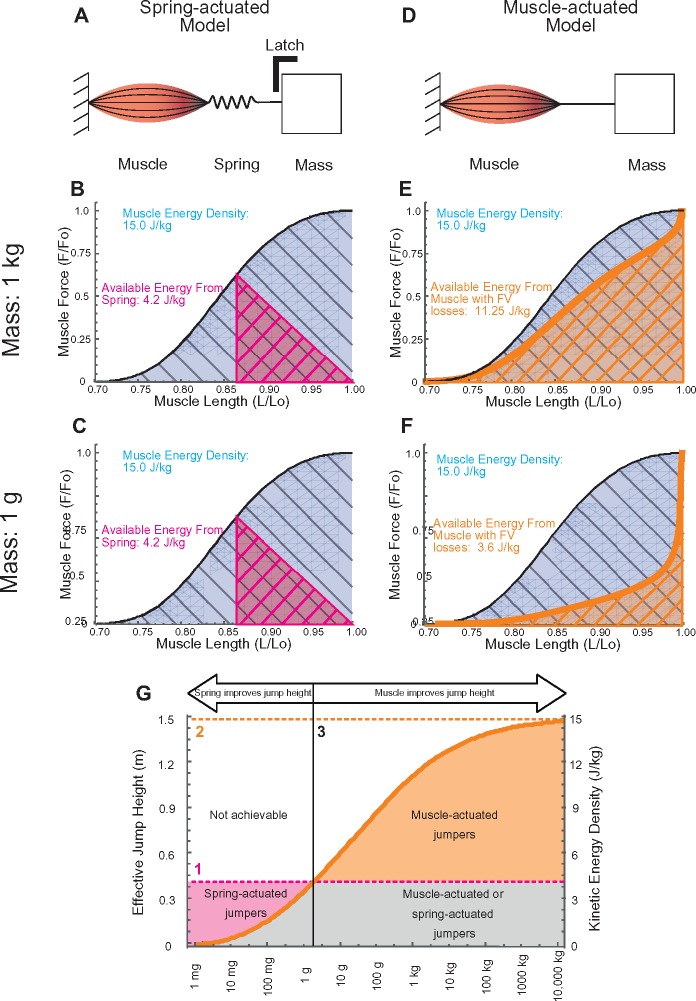
Two mathematical models compared the energetic costs and consequences of spring-actuated versus muscle-actuated jumps across animal sizes. In the spring-actuated model (**A**), the energy density of the muscle (J/kg), based on the integration of the muscle’s length–tension curve) is shown in blue and the energy density that could be stored in the spring (J/kg) is shown in magenta. Two example simulations are shown: a 1 kg spring-actuated system (B) and a 1 g spring-actuated system (**C**). As the system changes in size, the available energy density of the muscle does not change, and neither does the energy density of the spring, causing spring-actuated systems to store 28% of the available muscle energy—no matter the size of the mass. Consequently, jump height in spring-actuated systems is independent of size. The ratio of energy imparted to the mass (output energy, magenta) and energy available from the muscle (input energy, blue) is shown as a function of mass for spring-actuated systems in Fig. 1G (magenta dotted line). In the muscle-actuated model (**D**), the energy (in J/kg) available from the muscle is likewise shown in blue for a 1 kg (**E**) and a 1 g (**F**) mass simulation. The energy output versus length (the energy that accelerates the mass) is shown in orange. As the mass gets smaller, force–velocity properties reduce the muscle force, thus decreasing the output energy in the system, thus reducing the amount of energy that accelerates the mass. For the range of masses simulated, the effect of size on output energy (the energy that accelerates the mass) is shown by the orange solid line in Fig 1G. This reaches an asymptotic maximum possible jump height shown by the orange dashed line (2). Dashed lines 1 and 2 thus represent the alternative maximum jumping heights for spring-actuated and muscle-actuated jumpers, respectively. A size-related “crossover point” is evident (**G**, black line 3), such that animals smaller than this point can jump higher with a spring-actuated mechanism, whereas animals larger than this point can jump higher with a muscle-actuated mechanism.

Two kinetic models were used to compare size effects on the energy outputs of a muscle-actuated system and of a spring-actuated system. At the end of each actuator is a mass, *m* (Fig. 1). Both models are defined in terms of one fundamental parameter: Lo, the length of the muscle at which it generates its maximum force. The maximum muscle force, Fo, is proportional to muscle cross-sectional area or Lo^2^, and the mass is proportional to volume or Lo^3^, resulting in two model systems that isometrically scale in terms of Lo. Because most muscles work only on the ascending side of their length–tension curve, all simulations begin with the muscle at the length at which they generate maximum force (Zajac 1989). The energy density of the muscle was 15.0 J/kg, consistent with the properties of bullfrog plantaris muscles (Sawicki et al. 2015). The lengths of the muscle were varied to simulate accelerated masses from a range of 1 mg to 10,000 kg.

#### Latch-mediated spring-actuated model

The first model is a latched muscle-spring system (Fig. 1, left). To simplify the analysis, this muscle is assumed to be fully activated (i.e., no time-dependent activation parameters).

The muscle is a Hill-type muscle (Hill 1938) with force defined by:

(1) $$\mathrm{Muscle}\;\mathrm{Force}\;=\mathrm{Fo}\;*\;\mathrm{LT}\left(x\right)\;*\;\mathrm{FV}(\dot{x})$$

where x is the length of the muscle. The length–tension curve (*LT*) of the muscle is based on cat soleus, a representative of an “average” striated muscle based on parameters used in Rosario et al. (Zajac 1989; Rosario et al. 2016). *LT* is defined as:

(2) $$\mathrm{LT}(x)\;=\;e^{-{\left|\frac{{\left(\frac{x}{\mathrm{Lo}}\right)}^{B1}-1}{s}\right|}^{A1}}$$

where the constants are defined as: *A_1_* is 2.08, *B_1_* is −2.89, and *s* is 0.75.

Likewise, the force–velocity relationship (*FV*) of the muscle is:

(3) $$\mathrm{FV}(\dot{x})\;=\;\frac{1-\;\frac{\dot{x}}{\mathrm{Lo}\;{ɛ}_{\max}}}{\left(1+4\;\frac{\dot{x}}{\mathrm{Lo}\;{ɛ}_{\max}}\right)}$$

where ${ɛ}_{\max}$, the maximum strain rate of the muscle, is set equal to 10.0 s^−^^1^ (identical to the cat soleus muscle properties used in Rosario et al. 2016). The stiffness of the spring is such that the energy storage is optimized relative to the muscle. In other words, the spring stiffness maximizes the amount of energy that each spring could store relative to the muscle’s force and length. Rosario et al. (2016) reported the spring stiffness at which maximum energy storage occurs, which results from the interaction between muscle’s length–tension curve and the spring’s properties. For each simulation presented here, the spring stiffness was optimized to store the maximum amount of energy from each muscle (as calculated by Rosario et al. 2016).

Each simulation begins with the mass latched in place, the muscle maximally activated, and the spring not stretched. As the muscle slowly contracts, its ability to generate force decreases (Fig. 1B, thick black line) while the reaction force of the spring increases (Fig. 1B, magenta line). This continues until the force in the spring and the force in the muscle are equal. In other words, as the muscle moves along the ascending limb of the length–tension curve, the muscle’s generated force decreases and the reaction force of the spring increases. Equilibrium is reached when the muscle’s ability to generate force matches the force in the spring (Fig. 1B, intersection of thick magenta and thick black line), a similar analysis as in Rosario et al. (2016).

After the spring is loaded, the latch is released and the spring recoils, such that the stored energy is transferred completely to the mass. The mechanical energy available from the muscle (input energy) is calculated by integrating the length–tension curve of the muscle from the length at which it generates its maximum force to the length at which it generates the least force. The output mechanical energy is calculated by evaluating the kinetic energy (1/2 × mass × speed^2^) of the mass at the end of a simulation. Two output parameters were then calculated: the effective jump height if that animal were to jump purely vertically (jump kinetic energy/[body mass × *g*]), and the kinetic energy density of the jump (jump kinetic energy/mass).

#### Muscle-actuated model

The second model is a muscle-actuated system (Fig. 1, right).

The muscle properties for the second model are identical to those in the spring-actuated model, as are the scaling properties. Likewise, all the variable parameters are defined in terms of Lo, as they were for the spring-actuated model.

Each simulation begins with the muscle at the length at which it generates its maximal force (*Fo*) and as with the spring-actuated model, the muscle is assumed to reach full activation instantaneously. Force (and work) from the muscle then directly accelerates the animal’s body mass. As the mass accelerates, the muscle shortens, causing the force generated by the muscle to be altered by changing its position on both its length–tension and force–velocity curve. As with the first model, the input mechanical energy is calculated by integrating the length–tension curve of the muscle from its optimal length to its shortest force-producing length. The “input energy” thus represents the maximum energy that the muscle could generate, given the assumption that the muscle contracts isometrically (i.e., infinitely slowly). Note that the term “isometric” refers here to constant length, whereas throughout the rest of this manuscript we use the term “isometric” to refer to scaling (i.e., areas are proportional to Length^2^ and volumes to Length^3^). To avoid confusion, henceforth, we refer to “isometric” muscle contractions as “infinitely slow” to prevent confusion between the two uses of “isometric.” “Output mechanical energy” (i.e., the kinetic energy of the mass) is calculated by evaluating the kinetic energy (1/2 × mass × speed^2^) of the mass at the end of a simulation.

#### Interpretation of jump height data from the literature

From literature, we examined the distribution of animal jump heights relative to their body mass. We compiled data from 140 species of jumping animals from the literature: data from Alexander (1974); Brackenbury and Wang (1995); Burrows (2006, 2009, 2011, 2013, 2014); Burrows and Dorosenko (2014; 2015a; 2015b); Burrows et al. (2007); Burrows and Morris (2002, 2003); Burrows and Wolf (2002); Evans (1972); Burrows and Picker (2010); Patek et al. (2006); Picker et al. (2012); Schwaner et al. (2018); Sutton and Burrows (2011); and Moen et al. (2013). Launch speed (speed when they leave the ground) was compiled from these papers. We only included animals in the dataset if 10–20% body mass is devoted to muscles that directly or indirectly power jumps. This constraint was applied so that we could compare animals that would have approximately the same muscle-mass specific energy available for a jump. The kinetic energy (1/2 × body mass × take-off speed^2^) for each animal was calculated. We calculated the effective jump height with the assumption that the jumps are vertical (kinetic energy/[body mass × g]. We also calculated kinetic energy density of the jump (jump kinetic energy/body mass).

Jump height was estimated by taking the launch speed, *v*, of the data from the literature and estimating the jump height, *h*, assuming the animal jumps vertically.

The equation used to estimate jump height is:

(4) $$h\;=\;½{\;v}^{2}/g$$

From this equation, kinetic energy density is also calculated, which is:

(5) $$\text{Total kinetic energy }=\;½{\;m\;v}^{2}=\;m\;g\;h$$

(6) $$\text{Energy density }=\text{ kinetic energy}/\text{mass }=\;½{\;v}^{2}=\;g\;h$$

All papers included in the analysis measured jumps that started from a static position (a “squat” jump), and animals were only included if they have jumping muscles equivalent to 10–20% of their body mass.

## Results

In the spring-actuated model, neither input energy density (the maximum energy available from the muscle) nor output energy density (the energy that was transmitted to the mass) varies with size. Independent of size, each modeled muscle had an available energy density of 15.0 J/kg (Fig. 1, gray hatched areas). The independence of muscle size on input energy density is a consequence of a muscle’s maximum force being proportional to its cross sectional area (which scales with Lo^2^) while the distance that the muscle can apply force scales with Lo. The energy the muscle can generate is proportional to its force multiplied by the distance over which it can apply this force (Lo^2^ × Lo), and is thus proportional to Lo^3^. Mass is also proportional to Lo^3^, and consequently, the energy density (energy/mass) is a constant (Lo^3^/Lo^3^), consistent with previous predictions (Vogel 2005). Likewise, the output energy was also independent of size. The muscle begins each simulation at its optimal length whereas the spring begins each simulation with no tension; as the muscle contracts, its ability to generate force decreases (Fig. 1B, thick black line) while the reaction force of the spring increases (Fig. 1B, magenta line). This continues until the force in the spring and the force in the muscle are equal; that is, as the muscle length decreases, the muscle’s ability to generate force decreases. Meanwhile, as the spring lengthens, its force increases. Equilibrium is reached when the muscle’s ability to generate force matches the opposing force by the spring (Fig. 1B, intersection of thick magenta and thick black line). This arrangement results in a spring that can maximally store only 28% of the available input energy (4.2 J/kg) (Fig. 1B, compare the gray thatched area to the area under the magenta triangle). This 28% is for a spring that is of the optimal stiffness to store the maximum amount of energy relative to the muscle’s length–tension curve; a spring that is either more or less stiff than this would store less energy (Rosario et al. 2016). This 28% is also independent of animal size. Upon spring recoil, all of the energy stored in the spring is then transmitted to the mass.

As in the spring-actuated model, the input energy density of the muscle-actuated model (the maximum possible energy available from the muscle) does not vary with size; as the muscle gets smaller, the energy production capability decreases just as quickly as its mass, thus keeping the energy density of the muscle constant (15.0 J/kg for this simulation; Sawicki et al. 2015). As the system decreases in size, however, the output energy density (the energy that accelerates the mass) decreases quite precipitously. This is because, as the system gets smaller, reaching a given velocity of the mass requires a higher shortening strain-rate of the muscle. This reduces the muscle’s contractile force according to the force–velocity property of the muscle, and thus the muscle generates less mechanical energy, consistent with Zajac (1989) and Bobbert (2013). In the example in Fig. 1 E, a 1 kg mass is driven by a muscle with an energy density of 15 J/kg, but force–velocity losses cause the muscle to impart 25% less energy to the mass than would be imparted were the muscle allowed to contract extremely slowly (i.e., the reduction in force caused by the force–velocity property reduces the energy the muscle could generate by 25%), resulting in a density of energy output of 11.25 J/kg (the mass’s final speed is thus 4.7 m/s). In the contrasting example of a muscle that drives a 1 g mass (Fig. 1F), force–velocity properties reduce the muscle force even further than they do in the 1 kg case, allowing the muscle to generate a power density output of 3.6 J/kg (the mass’s final velocity is 2.7 m/s). So, as simulated jumping systems get smaller, the muscle-actuated model becomes less and less capable of driving a mass.

**Fig. 2:**
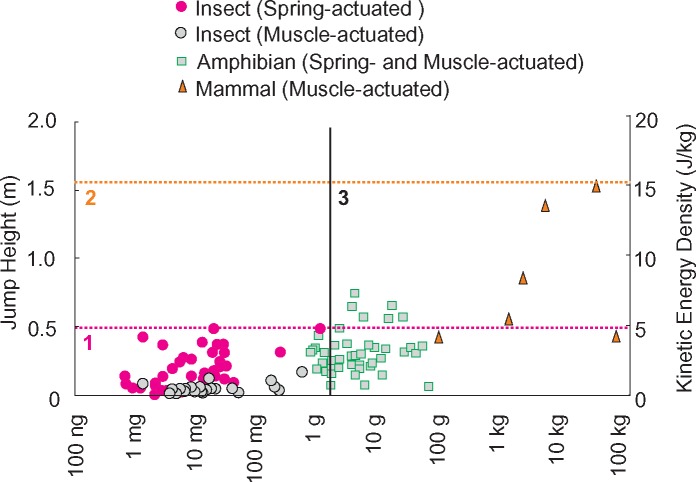
Jump heights and energy densities of the whole system for 142 species. Spring-actuated jumping insects are shown with gray circles with a black circumference, muscle-actuated insects are shown with filled magenta circles. Amphibians are shown in green squares (these use a combination of spring-actuated and muscle-actuated systems). Muscle-actuated jumping mammals are shown with orange triangles with black edges. Magenta dashed line (1): the jump height limit for purely spring-driven systems, estimated as 28% of the available muscle energy, as predicted by the simulations in Fig. 1. Orange dashed line (2): the jump height limit for purely muscle-actuated systems, estimated as 100% of the available muscle energy, as predicted by the simulations in 1. The black vertical line (3) represents the predicted “crossover point” between spring and muscle actuated systems. Data are from Alexander (1974); Brackenbury and Wang (1995); Burrows (2006); Burrows (2009, 2011, 2013, 2014); Burrows and Dorosenko (2015a, 2015b); Burrows et al. (2007); Burrows and Morris (2002, 2003); Burrows and Wolf (2002); Burrows and Dorosenko (2014); Evans (1972); Burrows and Picker (2010); Patek et al. (2006); Picker et al. (2012); Schwaner et al. (2018); Sutton and Burrows (2011); and Moen et al. (2013).

For masses >2.5 g (Fig. 1G), the modeled mass specific output of a muscle-actuated system is higher than that of a spring-actuated system. This is because the spring-actuated system, while having no loss in the ability of the muscle to generate force caused by velocity properties, is only able to store 28% of the energy that the muscle could produce (because of the effects illustrated in Fig. 1D). At masses <2.5 g, however, there is a “crossover point” where the force–velocity properties cause the muscles to have such low contractile forces that the amount of energy imparted to the mass by a muscle-actuated system is less than that of an equivalently sized spring-actuated system (Fig. 1G). This results in the limit shown by the dashed magenta line (Line 1) in Figs. 1G and 2. This analysis makes three predictions: (1) jump height for spring-actuated jumpers are independent of the animal’s size (consistent with Alexander 1995), (2) jump height for muscle-actuated jumpers increases with animal size (consistent with Alexander 1995 and Bobbert 2013), and (3) there is a crossover point above which muscle-actuated jumpers are able to jump higher than equivalently sized spring-actuated jumpers (in disagreement with Alexander 1995). The maximum jump height for the largest muscle-actuated jumpers will be approximately three times higher than that achievable by spring-actuated jumpers. These predictions can be non-dimensionalized as a relationship between the dimensionless kinetic energy of the mass (½ *m v*^2^)/(0.158 *Fo* Lo), and the dimensionless mass of a system (½ $m\;{\left(\mathrm{Lo}\;\dot{{ɛ}_{\max}}\right)}^{2}$) /(0.158 *Fo* Lo)(See Supplementary Materials).

To test these three predictions, the jump heights (equation 4) and energy densities (equation 6) were calculated for 140 species of animals, each with an equivalent percentage of their body mass devoted to jumping muscles (10–20%, Fig. 2). Consistent with Prediction 1, there appeared to be no effect of size on jump height for spring-actuated jumpers (Fig. 2, solid magenta circles). Consistent with Prediction 2, small muscle-actuated jumpers generated jump heights <0.2 m with the maximum jump height increasing as the muscle-actuated jumpers increase in size reaching a maximum at ∼10 kg; with the maximum jump heights of 1.54 m and 1.40 m observed by the domestic dog (*Canis familiaris*, 30–40 kg) and the rock wallaby (*Petrogale xanthopus*, 5–10 kg), respectively. Consistent with Prediction 3, the maximum jump height observed from a muscle-driven jumper (*C. familiaris*: 1.54 m) is 3.1 times larger than the maximum jump height observed from spring-actuated jumpers (0.49 m, either *Schistocerca gregaria*, Bennet-Clark 1975) or *Raphiophora vitrea*, Burrows 2014). This crossover point thus appears in the biological data as well as in the simulations. Due to the lack of robust phylogenies spanning the species illustrated in Fig. 2 (largely due to uncertainties in insect phylogeny), it was not possible to analyze these data quantitatively using phylogeny-based methods.

Considering Fig. 1G, the simulation’s prediction is that as body mass near the crossover point, jump height and energy density would increase. However, the frog data does not follow this pattern across body masses, with some having higher energy density than spring-actuated insects and some having lower (Fig. 2).

## Discussion

Spring-actuated systems are present in many biological systems, including mantis shrimp (Patek et al. 2004), alpheid shrimp (Ritzmann 1973), grasshoppers (Bennet-Clark 1976), fleas (Bennet-Clark and Lucey 1967; Sutton and Burrows 2011), froghoppers (Burrows 2003; Burrows 2006), fish (Van Wassenbergh et al. 2008; Longo et al. 2018), trap-jaw ants (Gronenberg 1995; Gronenberg 1996), and many others (Gronenberg 1996; Ilton et al. 2018). All of these systems accelerate relatively small masses. Frogs transition from spring-actuated jumps in smaller frogs to muscle-actuated jumps at larger sizes (Marsh 1994). Our theoretical analysis supports this observed pattern of biological variation. Based on our on energetics analysis, while spring-actuated systems have the capability to generate high levels of *power* density (up to millions of W/kg; Larabee et al. 2018), spring-actuated systems exhibit a finite *energy* density. Even under conditions that maximize the amount of energy stored in a spring, a spring-actuated system can only store ∼30% of the energy that a muscle can produce (presented here and in Rosario et al. 2016). This limit gives larger spring-actuated system a lower energy output than an equivalently sized muscle-actuated system. As systems decrease in size (<1 g), however, force–velocity effects of the muscle to generate force reduce the amount of mechanical energy the muscles can generate. If velocity effects reduce muscle output by >70%, as would happen for smaller animals, a spring-actuated system becomes more favorable because it can generate more kinetic energy than an equally sized muscle-actuated system. Consequently, instead of all jumping animals approaching the same possible jump height (as predicted by Borelli 1680) there are, instead two different energetic limits for jump height: one for muscle-actuated jumpers and one for spring actuated jumpers; with the maximum spring actuated height being approximately one-third as high as the maximum height possible for muscle-actuated jumpers. Frogs, existing in an intermediate size range, have a mechanism that combines both muscle-actuation and spring-actuation, providing jump heights that are intermediate between the two. There is thus a size related cross-over point: animals larger than this would jump higher with a muscle-actuated jump, while animals smaller than this would jump higher with a spring-actuated jump.

In our simulations, the spring could maximally store 28% of the energy available from the muscle, which was modeled on the cat soleus muscle. Depending on the exact length–tension properties of other muscles, initial placement of the springs relative to the length–tension curve, and the stiffness of the spring, this number could vary. Nevertheless, for any system in which a muscle slowly loads a spring, there will be two reasons that the muscle cannot store 100% of its maximum available energy in a spring: (1) at long muscle lengths, the spring cannot resist the movement with all of the force the muscle can generate (the upper right area of the length–tension curve, Fig. 1B and C, blue) and (2) at short muscle lengths, the muscle cannot generate enough force to stretch the spring further (the lower left area of the length–tension curve, Fig. 1B, C, blue). For length–tension curves that reflect standard vertebrate muscle, this will constrain the muscle to storing only about one-third of its available energy in a spring. If a system is such that force–velocity induced reductions of available work are less than length–tension/spring induced reductions of available work, then muscle-actuation will provide more energy than spring-actuation.

There are a few cases in which a muscle driven jumper reached higher jump heights than spring driven jumpers and operated with slightly higher energy densities. Such deviations from our predictions likely arise from natural biological variation related to the diverse ecology of the organisms and potential variation in the relative importance of this behavior to the animals’ biology. We found that jumpers that were at masses >1 kg produced jump heights that were approximately three times higher than the spring actuated jumpers. We considered these jumpers to be muscle-actuated jumpers because to the authors’ knowledge there is no documentation of organisms >1 kg that are primarily spring-actuated. Taken all together, these data support our modeling results that there are two size-specific mechanisms to maximize jump height. Organisms that operate at intermediate body sizes likely occupy an area near the crossover point where the two described mechanisms (spring- versus muscle-driven) are difficult to distinguish. The animal data show that this zone is largely occupied by the anurans, which span body masses from 1 g to slightly below 1 kg (Fig. 2, squares). Frogs provide an interesting test case given that the mechanism used for spring actuation does not allow for jumps that are solely spring driven; instead they often rely on the contribution of proximal hind limb muscles which have little to no elastic structures (Olson and Marsh 1998; Astley 2016). In addition, comparative analyses of jump performance have suggested that the utilization of spring actuation varies significantly within this group and that role of spring actuation is likely diminished in larger species (Mendoza 2018). Moreover, the jump speeds and elastic recoil rates of frogs give them an opportunity for muscles and springs to actuate the jump simultaneously, resulting in dynamics that create an intermediate between spring and muscle actuation.

There are a number of other features of integrated biological systems that can affect the actualized energy or power density of jumps which were not considered here which would affect the location of the cross over point we describe. For instance, this analysis does not incorporate the role of countermovements prior to jumping, which can also be used by larger animals to also increase jump height (Zajac 1993; Alexander 1995). Likewise, jumping by extending limb joints results in increasing effective mechanical advantage (EMA) of the limb extensor muscles throughout the motion (Astley and Roberts 2012; Olberding et al. 2019). Limb morphology and properties of the muscle and body mass determine the range and rate of change in EMA throughout the jump, which influences the acceleration of muscle contraction, associated changes in force along the force–velocity curve and the total work done (Olberding et al. 2019). Lastly, moment-arm dynamics could also affect the energy available in a muscle-actuated system (Galantis and Woledge 2003). These effects should only impact larger muscle-actuated jumping animals by increasing or decreasing their jump height depending on the nature of the lever system. None of these effects, however, would affect the fundamental conclusion that the interaction of length–tension and force–velocity properties creates a size related “crossover point” for spring-actuated and muscle-actuated jumpers: systems smaller than this point can jump higher with a latch-mediated spring actuated system, while larger systems can jump higher with a purely muscle-actuated system. These effects would only move the location of the crossover point, but would not eliminate it. These crossover points have been shown for muscle-actuated lever arm systems (Galantis and Woledge 2003) and for motor driven systems (Ilton et al. 2018), but this is, to the authors’ knowledge, the first illustration of this point in comparing springs and muscles in biological organisms.

The muscle-actuated model begins when the muscle has already reached peak force. Actual muscle requires time to fully activate muscle fibers and develop peak force. Depending on the properties of a latch in a muscle-actuated movement, activation dynamics could greatly alter the work done by the muscle (Olberding et al. 2019). If the force–velocity relationship is scaled to the level of activation, then shortening occurring before the muscle is fully activated will be relatively small. As activation continues, the strain rate of the muscle will increase, causing the force–velocity properties of the muscle to decrease the resulting contractile force. Overall, the lower peak force reached by the muscle will reduce the total work, such that the crossover point is shifted to somewhat larger sizes. Likewise, the presented model assumes that the muscle starts at its optimal length and that the length–tension curve does not change as a function of muscle activation. Both of these are assumptions that do not apply to all systems. Before jumping frogs begin loading their elastic mechanism, their muscles sometimes start contracting at lengths exceeding the optimal muscle length (Azizi 2014), and the length–tension curves change as a function of activation (Holt and Azizi 2016). Both of these issues should influence the mass at which the crossover point between spring and muscle-driven systems occurs, but neither would change the fact that there is a crossover point.

Special consideration should be given to the loss of energy to gravitational potential energy as animals extend their legs (Scholz et al. 2006). As the animal extends its legs, the work is directed into kinetic energy (1/2 *mv*^2^) and gravitational potential (*mgh*). In insects and smaller frogs the gravitational potential during take-off is small, ranging from 1% to 5%. In the case of larger animals (such as a cat and a dog), however, applying this effect to legs that are 20–30 cm long could underestimate muscle work by 15–20% (i.e., the dog in the dataset was able to reach a height of 1.5 m, but it lost about 0.3 m of effective jump height to leg extension before it “took-off,” meaning that the muscles generated enough work to lift it 1.8 m instead of 1.5 m). Consequently, while the literature shows dogs generating 3.1 times as much kinetic energy density as spring-actuated jumpers, this gravitational potential would cause dogs to generate 3.7 times as much work as a spring-actuated jumper. This is still within the range of output predictions from the models presented here (which predict that larger muscle-actuated jumpers would generate 3.6 times as much energy as would larger spring-actuated jumpers). This added complexity would not change the fundamental point that larger animals, using a muscle-actuated system, are able to jump several times higher than they would using a spring-actuated system.

The differential scaling of energy limits imposed by FL effects in spring-driven systems and FV effects in muscle-driven systems creates a crossover size below which spring-driven jumping is best and above which muscle-driven jumping is best. Although our models are necessarily quite simple, a crossover is inescapable in any biological system based on our understanding of FL and FV effects in muscles and biological springs. We have presented biological data that demonstrate this trend, in general, and have discussed a number of additional considerations that may change the exact size at which this crossover occurs. Exactly how these different potential manipulations quantitatively interact within an integrated organism is a question for further work, but it is worth mentioning that the bush baby (*Galago senegalensis*; Aerts 1998) uses many of the discussed additional mechanisms and generates spectacularly high jumps (as high as 2 m). The analysis presented here, however, is intended to answer the fundamental question brought up from our introduction: “Why don’t large animals use spring-actuation to jump?”. The answer is because they can jump a lot higher without spring actuation.

## Authors’ contributions

G.P.S., E.A., and S.N.P. contributed to the conceptualization of the study. G.P.S., E.M., and S.J.L. contributed to data curation. G.P.S., J.P.O., and M.I. contributed to modeling and software. G.P.S., E.A., S.N.P., E.M., S.J.L., J.P.O., and M.I. contributed to the writing of the manuscript.

## Funding

This work was funded by the Royal Society (UF130507) and the Army Research Office under grant number W911NF-15-1-0358.

## Supplementary Material

icz145_Supplementary_Data

## Notes

From the symposium “Playing with Power: Mechanisms of Energy Flow in Organismal Movement” presented at the annual meeting of the Society for Integrative and Comparative Biology, January 3–7, 2019 at Tampa, Florida.
