# Corrigendum to: Why do Large Animals Never Actuate Their Jumps with Latch Mediated Springs? Because They can Jump Higher Without Them

**DOI:** 10.1093/icb/icz149

**Published:** 2019-09-26

**Authors:** Gregory P Sutton, Elizabeth Mendoza, Emanuel Azizi, Sarah J Longo, Jeffrey P Olberding, Mark Ilton, Sheila N Patek

**Affiliations:** 1 School of Life Sciences, University of Lincoln, Lincoln, UK; 2 School of Biological Sciences, University of California, Irvine, CA, USA; 3 Biology Department, Duke University, Durham, NC; 4 Department of Physics, Harvey Mudd College, Claremont, CA

*Integrative and Comparative Biology*, doi:10.1093/icb/icz145

The affiliation of author Mark Ilton was incorrectly provided as “Harvey Mudd University”. This has been corrected to “Harvey Mudd College”.

Figure 2 has also been updated.

