# Erratum to: Why do Large Animals Never Actuate Their Jumps with Latch-Mediated Springs? Because They can Jump Higher Without Them

**DOI:** 10.1093/icb/icaa008

**Published:** 2020-03-16

*Integrative and Comparative Biology*, volume 59, number 6, pp. 1609–1618

In the original manuscript the authors inadvertently included 24 data points of unpublished anuran data in Figure 2. We have now enclosed a Figure 2 that removes all of the unpublished data, and thus now includes a total of 116 species (anuran and non anuran). The published anuran data remaining in Figure 2 is from Moen et al., 2013 (cited in the bibliography). We would like to apologize for this oversight.

Secondly, the bibliography entry for Mendoza E., 2018, should be updated to be a thesis from Oklahoma State University, not UC Irvine, as stated.

Lastly, M. Ilton’s association should be listed as ‘Harvey Mudd College, Claremont, CA’, not ‘Harvey Mudd University, Claremont, CA’.

