# Supplementary material for: Why do Large Animals Never Actuate Their Jumps with Latch-Mediated Springs? Because They can Jump Higher Without Them
Source: Integr Comp Biol. 2019 Aug 9;59(6):1609–18. doi: 10.1093/icb/icz145 (PMC6907395; doi:10.1093/icb/icz145)
Supplement: icz145_Supplementary_Data [file icz145_supplementary_data.docx]

Supplemental Material:

The data presented in figure 1G can be re-plotted to reflect two dimensionless parameters: a) a non-dimensionalized energy output, $\hat{E}_{\mathrm{out}}$, and b) a non-dimensionalized mass, $\hat{m}$.

The non-dimensionalized energy output, $\hat{E}_{\mathrm{out}}$, is the ratio of the amount of kinetic energy in in the system ($\frac{1}{2}mv^{2}$) and the maximum energy$,E_{\max}$, output that the muscle could generate during a quasi-static muscle contraction . In the muscle model presented here, $E_{\max}=0.158 F_{0}L_{0}$. Consequently, the first of these parameters, $\hat{E}_{\mathrm{out}}$ is thus:

$\hat{E}_{\mathrm{out}}=\frac{\frac{1}{2}mv^{2}}{0.158 Fo Lo}$

The non-dimensionalized mass, $\hat{m}$, is derived from solving a dimensionless representation of Newton’s second law: $\hat{F}=\hat{m} \hat{a}$. To solve for the normalized mass, we need the ratio of the normalized Force ($\hat{F}$) and the normalized acceleration $\hat{a}$. The normalized force, $\hat{F}$, is the ratio of the muscle force F and the maximum muscle force Fo.

$\hat{F}=\frac{F}{Fo}$

Calculating the normalized acceleration, $\hat{a}$, requires a normalized velocity, $\hat{v}$, and a normalized time, $\hat{t}$, based on the maximum contractile velocity of the muscle. The normalized velocity, $\hat{v}$, is the velocity of the system divided by the maximum possible velocity at which the muscle can generate force:

$\hat{v}$ = $\frac{V}{Lȯ_{max}}$

And the normalized time, $\hat{t}$, is the ratio of time and the time it would take for the system to move one muscle length at the muscle’s maximum contractile velocity:

$\hat{t}= \frac{t}{\frac{Lo}{Lo ̇_{max}}}=t ̇_{max}$

The normalized acceleration, $\hat{a}$, is thus the normalized velocity, $\hat{v}$, divided by the normalized time, $\hat{t}$:

$\hat{a}= \frac{\hat{v}}{\hat{t}}= \frac{\frac{V}{Lȯ_{max}}}{t ̇_{max}}$ = $\frac{V}{Lo t {̇_{max}}^{2}}$

The normalized mass, $\hat{m}$, is the normalized Force, $\hat{F}$, divided by the normalized acceleration,$\hat{a}$:

$\hat{m}= \frac{\hat{F}}{\hat{a}} = \frac{\frac{F}{Fo}}{\frac{V}{Lo t ̇_{max}}}$ = $\frac{F t}{V} \frac{Lo {̇_{max}}^{2}}{Fo}$

The ratio, $\frac{F t}{V}$, is a another expression for the mass of the system, m, thus yielding the final expression for dimensionless mass:

$\hat{m}$= $m \frac{Lo {̇_{max}}^{2}}{Fo}$

Plotting $\hat{E}_{\mathrm{out}}$ vs $\hat{m}$ (supplemental figure, thick orange line), shows two regimes of behavior for the normalized kinetic energy output. First, for low organism mass, $\hat{E}_{\mathrm{out}}$ increases as a function of increasing  $\hat{m}$. In this regime, the kinetic energy output is determined by the maximum contractile velocity of the muscle and therefore $\hat{E}_{\mathrm{out}} is proportional to \frac{\frac{1}{2}m Lo {̇_{max}}^{2}}{0.158 F_{0}}$(i.e. it follows the dashed blue line in the supplemental figure. Secondly, at higher organism mass, the inertial mass of the organism is much greater than the characteristic mass determined from muscle performance, and the muscle contracts slowly. In this limit, the kinetic energy output approaches  $E_{\max}$, which gives  $\hat{E}_{\mathrm{out}}\approx1$ (i.e. asymptotically approaching the orange dashed line). In comparison, for spring-actuated systems, $\hat{E}_{\mathrm{out}}$ is constant (supplemental figure, magenta line). By knowing the maximum strain rate of the muscle driving the behaviour, this analysis can show where, on this dimensionless space, an organism exists predicting whether it would be more efficient for the organism to generate a behaviour with a muscle-actuated system (orange) or a spring-actuated system (purple). In addition this predicts how the output velocity of this behaviour will scale with size.


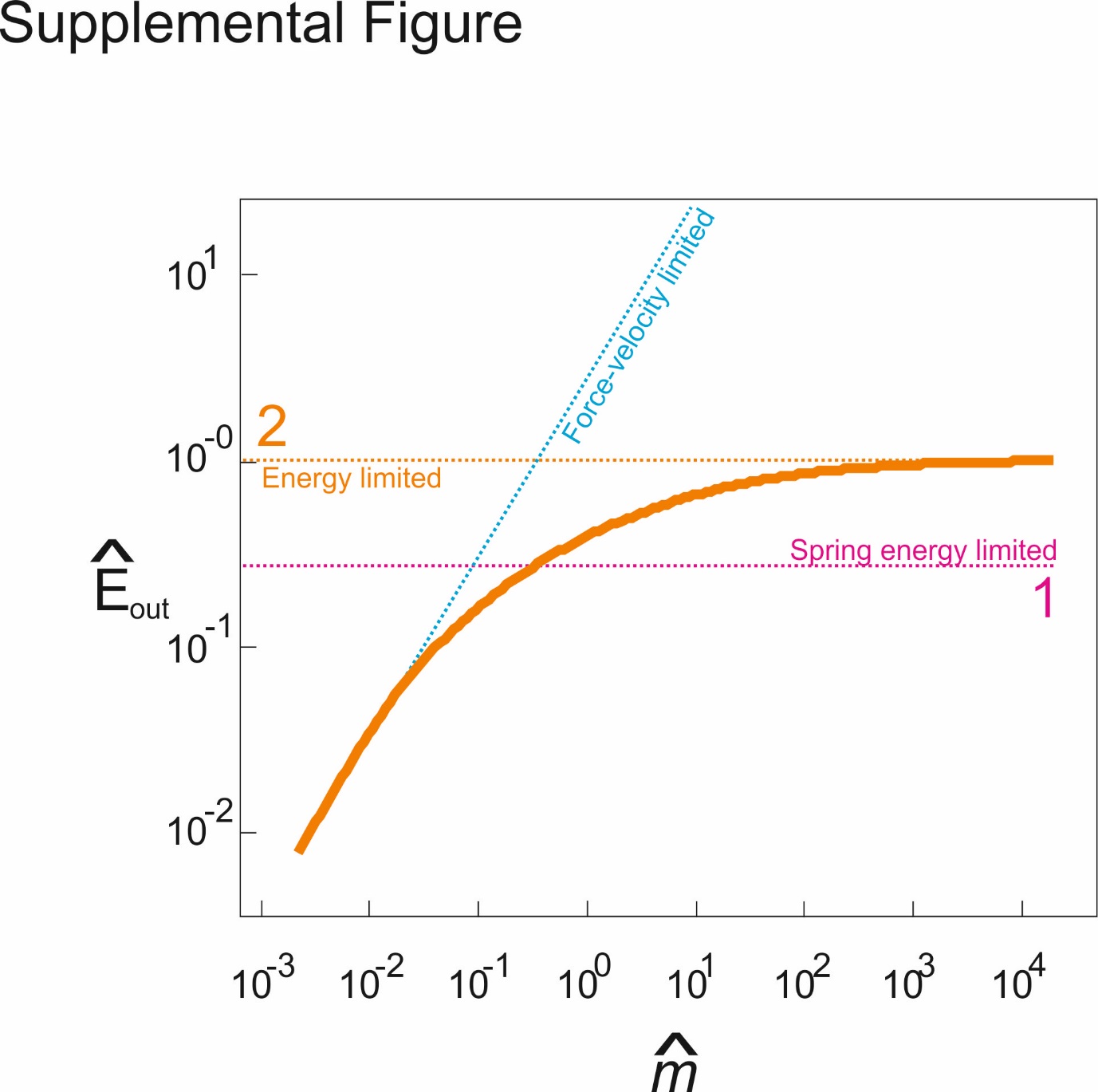


Supplemental Figure: Data from Figure 1G plotted in terms of normalized Energy,$\hat{E}_{\mathrm{out}}$ , and normalized mass, $\hat{m}$(thick orange line). The limits shown on figure 1G for muscle-actuated systems and spring-actuated systems are shown with the dashed orange and dashed magenta lines, respectively. At small values of normalized mass, limitations of energy output caused by the force-velocity property of muscle causes$\hat{E}_{\mathrm{out}}$ and $\hat{m}$ to be proportional (following the dashed blue line). As normalized mass gets larger, the output energy becomes limited by the amount of energy available in the muscle; and, consequently, asymptotically approaches 1.
